# Repression of CD24 surface protein expression by oncogenic Ras is relieved by inhibition of Raf but not MEK or PI3K

**DOI:** 10.3389/fcell.2015.00047

**Published:** 2015-08-05

**Authors:** Nikitha K. Pallegar, D. Craig Ayre, Sherri L. Christian

**Affiliations:** Department of Biochemistry, Memorial University of NewfoundlandSt. John's, NL, Canada

**Keywords:** oncogenic Ras, CD24, PI3K, Raf, gene expression, protein expression

## Abstract

CD24 is a dynamically regulated cell surface protein. High expression of CD24 leads to progression of lung, prostrate, colon, and pancreatic cancers, among others. In contrast, low expression of CD24 leads to cell proliferation and metastasis of breast cancer stem cells (BCSCs). Activating mutations in Ras are found in 30% of all human cancers. Oncogenic Ras constitutively stimulates the Raf, PI3K, and Ral GDS signaling pathways, leading to cellular transformation. Previous studies have shown that expression of oncogenic Ras in breast cancer cells generates CD24^−^ cells from CD24^+^ cells. However, the molecular mechanisms involved in the generation of CD24^−^ cells were not determined. Here, we demonstrate that oncogenic Ras (RasV12) expression suppresses CD24 mRNA, protein, and promoter levels when expressed in NIH/3T3 cells. Furthermore, activation of only the Raf pathway was sufficient to downregulate CD24 mRNA and protein expression to levels similar to those seen in with RasV12 expression. In contrast, activation of the PI3K pathway downregulated mRNA expression with a partial effect on protein expression whereas activation of the RalGDS pathway only partially affected protein expression. Surprisingly, inhibition of MEK with U0126 only partially restored *CD24* mRNA expression but not surface protein expression. In contrast, inhibition of Raf with sorafenib did not restore *CD24* mRNA expression but significantly increased the proportion of RasV12 cells expressing CD24. Therefore, the Raf pathway is the major repressor of CD24 mRNA and protein expression, with PI3K also able to substantially inhibit CD24 expression. Moreover, these data indicate that the levels of CD24 mRNA and surface protein are independently regulated. Although inhibition of Raf by sorafenib only partially restored CD24 expression, sorafenib should still be considered as a potential therapeutic strategy to alter CD24 expression in CD24^−^ cells, such as BCSCs.

## Background

CD24 is a cell surface protein that has various and diverse roles in cell adhesion and signaling, B lymphocyte and neuronal development, autoimmune diseases, and cancer (Fang et al., 2010). In many cancers, such as colorectal, pancreas, and lung, high expression of CD24 is associated with enhanced invasiveness and proliferation (Kristiansen et al., 2004). In stark contrast, forced expression of CD24 in breast cancer cells that have low CD24 expression (CD24^−^) leads to decreased cell proliferation (Ju et al., 2011). Breast cancer cells that have low expression of CD24 in combination with high expression of CD44 (CD24^−^/CD44^+^) and high aldehyde dehydrogenase (ALDH) activity are known as breast cancer stem cells (BCSCs) (Al-Hajj et al., 2003; Douville et al., 2009). BCSCs exhibit increased invasiveness and proliferation (Sheridan et al., 2006), and tend to be radiation resistant (Phillips et al., 2006). Tumors with high percentage of BCSCs generally have a worse prognosis (Kristiansen et al., 2003) since as few as 1 × 10^3^ BCSCs can regenerate an entire tumor including both CD24^−^ and CD24^+^ cells (Yan et al., 2013).

It has been shown that CD24 expression is dynamically regulated in both CD24^+^ and CD24^−^ breast cancer cells, with CD24^−^ cells gaining CD24 expression and *vice versa* (Meyer et al., 2009). This dynamic regulation occurs both *in vitro* and *in vivo* and is associated with changes to the invasive phenotype (Meyer et al., 2009). Moreover, it has been previously shown that CD24^−^/CD44^+^ stem like cells can be generated from CD24^+^/CD44^−^ cells after activation of the oncogenic Ras pathway (Morel et al., 2008). However, the mechanism underlying this regulation was not established.

*CD24* expression can be regulated transcriptionally or post-transcriptionally. Transcriptional regulation of *CD24* varies widely depending on the cell type. For example, the *CD24* promoter contains binding sites for SP-1, which promotes transcription of *CD24* in multiple sclerosis (Wang et al., 2012) and NFAT5 sites, which promote transcription of *CD24* in T-cells in response to hypertonicity (Berga-Bolaños et al., 2010). In contrast, the estrogen receptor (ER) represses *CD24* transcription in ER-positive breast cancer cells (Kaipparettu et al., 2008). The TWIST transcription factor family can upregulate or downregulate *CD24* expression, with TWIST1 shown to downregulate *CD24* transcription in BCSCs (Vesuna et al., 2009), and TWIST2 promoting *CD24* transcription in human hepatocarcinoma (Liu et al., 2014). In addition, the *CD24* promoter region contains a negative regulatory element located −983 to −1996 upstream of the transcription start site (TSS) that can repress *CD24* transcription via an unidentified transcription factor (Pass et al., 1998). Bioinformatic analysis using UCSC genome browser (Kent et al., 2002) reveals a CpG island between −828 to +430 bp, relative to the TSS and enhanced methylation of the *Cd24* promoter has been associated with decreased expression in glioblastoma cells (Fukushima et al., 2007) and diseased conjunctiva (Riau et al., 2011). Post-transcriptionally, the miR34a miRNA has been shown to repress *CD24* mRNA expression via the 3′ untranslated region (Muppala et al., 2013).

Ras is an oncogene with mutations present in approximately 30% of all human cancers (Schubbert et al., 2007) and has been shown to repress CD24 surface expression (Morel et al., 2008). Ras activates numerous signaling pathways, including the Raf, RalGDS, and phosphoinositide-3-kinase (PI3K) pathways to promote a myriad of cellular functions such as cell proliferation, cell transformation, and cell survival (McCubrey et al., 2007). Activation of RalGDS leads to activation of the RalA GTPases which leads to the subsequent activation of phospholipase D (PLD) to promote vesicle formation and membrane trafficking through the golgi (Feig, 2003). Activation of the Raf kinase leads to the phosphorylation and activation of MEK1/2 (MAP2K; mitogen-activated protein kinase kinase), which subsequently phosphorylates and activates the extracellular signal-regulated kinase (ERK1/2). The Raf/MEK/ERK pathway primarily regulates proliferation and apoptosis (Aksamitiene et al., 2012). Activation of PI3K leads to phosphorylation of phosphotidylinositol phospholipids that recruit and promote the activation of Akt by the PDK1 (phosphoinositide-dependent kinase 1) and PDK2 (phosphoinositide-dependent kinase 2) kinases. The PI3K/Akt pathway promotes cell survival, growth and metabolism in addition to regulating cell migration (Aksamitiene et al., 2012). The PI3K pathway can also be activated independently of Ras activation and substantial cross-talk between the Raf and PI3K pathways has been established (Aksamitiene et al., 2012).

Despite evidence that overexpression of oncogenic Ras can repress CD24 surface expression (Morel et al., 2008) the mechanism for this regulation of CD24 is not known. Here, we examined the regulation of CD24 mRNA, protein, and promoter levels in a model of oncogenic Ras activation. We found that oncogenic Ras can directly repress CD24 mRNA, protein and promoter activity. We further examined the pathways regulated by Ras to show that either the PI3K or Raf pathways can repress CD24 expression. Surprisingly, inhibition of Raf but not MEK or PI3K significantly increased CD24 surface expression.

## Methods

### Cells and treatments

NIH/3T3 cells expressing the empty pBabe vector (control), or the pBabe vector containing RasV12, RasV12S35, RasV12G37, or RasV12C40 were gifts from Dr. Kensuke Hirasawa, Memorial University of Newfoundland (Battcock et al., 2006). All cell lines were maintained in high-glucose Dulbecco's Modified Eagle Medium (DMEM) (Life Technologies Co., Burlington, ON) supplemented with 10% fetal bovine serum (FBS), 1% anti-mycotic/antibiotic, 1% sodium pyruvate (complete media) and maintained at 37°C in 5% CO_2_. Control and RasV12 cells (6 × 10^4^ – 2 × 10^5^ cells/plate) were seeded in 35 and 60 mm plates. At 50–60% confluency, cells were treated with the DMSO vehicle control, 20 μM U0126 (Calbiochem-Millipore, Billerica, MA), 5, 10, or 20 μM sorafenib (Santa Cruz Biotechnology Inc, Santa Cruz, CA, USA), or 100 μM LY294002 (Calbiochem), as indicated, in complete media.

### RNA isolation and RT-PCR

RNA was extracted from cells using TRIzol (Life Technologies) following the manufacturer's protocol followed by DNase treatment using the Turbo DNA-free kit (Life Technologies). RNA (500 ng) was reverse transcribed to cDNA using MMLV-RT (Life Technologies) with random hexamers, and then amplified with Taq DNA polymerase (Norgen Biotek, Thorold, ON) using the primers shown in Table 1. The PCR products were visualized on 1% agarose gel after staining with ethidium bromide.

**Table 1:** **Primers used**.

**Gene/Region**	**Sequence (5^′^−3^′^) of forward (F) and reverse (R) primers**	**Efficiency**	**Amplicon size**
**RT-PCR**			
*CD24*	F -CTT CTG GCA CTG CTC CTA CC R -AAC AGC CAA TTC GAG GTG GAC	N/A	300 bp
*RPLP0*	F -CGG CCC GTC TCT CGC CAG R -CAG TGA CCT CAC ACG GGG CG	N/A	448 bp
*H*-*Ras*	F -ATG ACG GAA TAT AAG CTG GTG R -TCA GGA GAG CAC ACA CTT GCA	N/A	570 bp
**RT-qPCR**			
*CD24*	F -ACT CAG GCC AGG AAA CGTCTCT R -AAC AGC CAA TTC GAG GTG GAC	1.07	109 bp
*RPLP0*	F -TCA CTG TGC CAG CTC AGA AC R -AAT TTC AAT GGT GCC TCT GG	1.03	101 bp
**PROMOTER AMPLIFICATION**			
−688/-1	F -GTT GGA TGC TCC CGG GTA TGG R -GGA GCG CGG CCG GCC GGC GG	N/A	688 bp

### Quantitative RT-PCR (RT-qPCR)

RT-qPCR was performed in triplicate using Maxima SYBR Green qPCR Master Mix (2X) (Thermo Fisher Scientific, Waltham, MA, USA) for *CD24* or *RPLP0* using the primers shown in Table 1 with the Eppendorf RealPlex^2^ Real Time PCR machine. Relative *CD24* mRNA levels were normalized using *RPLP0* and calculated using the ΔΔCt equation (Livak and Schmittgen, 2001).

### Flow cytometry

Single cell suspensions (0.2–0.5 × 10^6^ cells for NIH/3T3 derived cells) were obtained by scraping cells from plates into FACS buffer [1% heat inactivated FBS in phosphate-buffered saline (PBS, 1.86 mM NaH_2_PO_4_.H_2_O, 8.41 mM Na_2_HPO_4_, 150 mM NaCl)]. Cells were incubated with 0.5 μg anti-CD24 (M1/69), or Rat IgG2a κ isotype control, conjugated to APC (eBioscience, San Diego, CA, USA) for 30 min on ice, followed by three washes with FACS buffer then fixed in 4% paraformaldehyde. Data was collected with a FACS Calibur (BD Biosciences, Mississauga, ON, Canada) and analyzed using FlowJo software v10.0.5 (Ashland, OR, USA).

### Western blot analysis

Cells were washed with PBS and then lysed in RIPA buffer (50 mM Tris-HCl (pH 7.6), 0.02% sodium azide, 0.5% sodium deoxycholate, 0.1% SDS, 1% NP-40, 150 mM NaCl) containing 1 μg/ml aprotinin, 1 mM PMSF and 1X HALT phosphatase inhibitor cocktail (Thermo Fisher Scientific). Protein concentration was determined by Bicinchoninic acid (BCA) protein assay (Thermo Fisher Scientific). The samples were subjected to 10% SDS polyacrylamide gel electrophoresis and transferred to nitrocellulose membrane, which was blocked in 5% skimmed milk in Tris-buffered saline (10 mM Tris base, 150 mM NaCl, pH7.5) and 0.05% Tween-20 (TBST). Primary antibodies to detect phosphorylated ERK (Cat #9101), phosphorylated Akt (Cat #9271), total ERK (Cat #4695), and total Akt (Cat #9272) were obtained from Cell Signaling Technologies, Inc (Danvers, MA, USA). Secondary antibodies were obtained from Santa Cruz Biotechnology Inc. Immobilon Western Chemiluminescent HRP substrate was used for detection (EMD Millipore, Darmstadt, Germany) followed by imaging with the ImageQuant LAS 4000 (GE Healthcare, Morgan Boulevard, Baie d'Urfe Quebec, Canada).

### CD24 promoter analysis

Genomic DNA was isolated from a male C57BL/6N mouse liver using the Genomic DNA isolation kit (QIAGEN, Germantown, MD, USA). The *CD24* promoter region from −688 to −1 from the TSS was amplified from genomic DNA with the GC-Rich PCR system (Roche, Basil, Switzerland) using the primers indicated in Table 1. The promoter was cloned into the HindIII and BglII sites of the pGL4.17 vector (Promega, Madison, USA). The deletion constructs −469 to −1, −357 to −1, and −168 to −1 were generated using the Erase-a-base kit (Promega) according to the manufacturer's instructions. All sequences were verified by sequencing at The Centre for Applied Genomics (Toronto, ON, Canada).

Control or RasV12 cells (3 × 10^4^ cells/well in 24-well-plates) were transfected with 1 μg of the pGL4.17 vector with or without the *CD24* promoter regions and 6.66 ng pRL-SV40 vector (Promega) using 2.5 μl Superfect transfection reagent (Qiagen), following the manufacturer's instructions. After 24 h, cells were lysed with 1X Passive Lysis Buffer and Firefly and Renilla Luciferase activity were measured using the Dual-Luciferase Reporter Assay kit (Promega).

## Results

### RasV12 downregulates CD24 mRNA and surface protein expression

**Figure 1:**
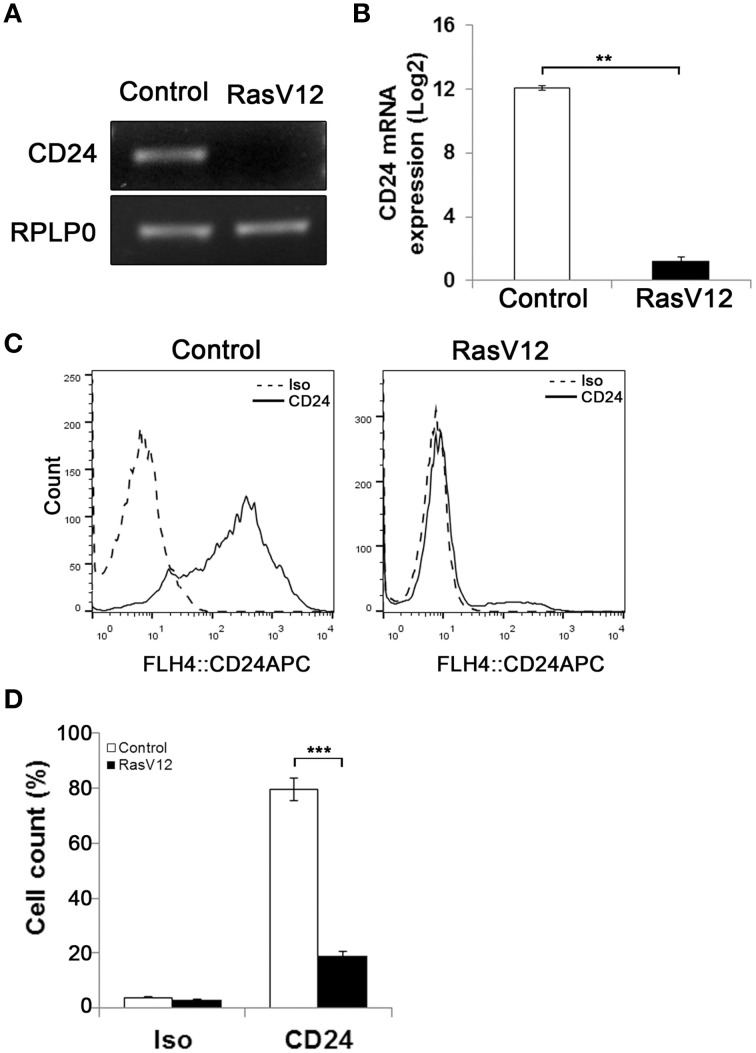
**Oncogenic Ras downregulates CD24 expression in NIH/3T3 cells**. *CD24* mRNA expression in vector control (Control) and RasV12 cells was determined by **(A)** RT-PCR and **(B)** RT-qPCR. *RPLP0* was used as the loading and normalization control. *CD24* mRNA levels shown as mean ± s.e.m. **(C)** Surface CD24 protein was determined by flow cytometry in Control and RasV12 cells. One representative histogram of isotype (Iso) and CD24-stained cells is shown. **(D)** Quantification of CD24 surface protein expression as mean ± s.e.m percentage of CD24^+^ cells. Significance was determined by Student's *t*-test, *n* = 3, ^**^P < 0.01; ^***^P < 0.001.

Previous studies have shown that activation of oncogenic Ras leads to generation of CD24^−^/CD44^+^ stem-like cells from CD24^+^/CD44^low^ cells (Morel et al., 2008). To analyze the regulation of CD24 expression by oncogenic Ras we used a model system in which constitutively active H-Ras, containing a G12 to V12 mutation (RasV12), was stably expressed in the mouse embryonic fibroblast NIH/3T3 cell line (Christian et al., 2009). The level of *CD24* mRNA expression in NIH/3T3 cells stably transfected with empty vector (control cells) or containing the constitutively active Ras gene was analyzed by RT-PCR and RT-qPCR (Figures 1A,B). We observed a clear suppression of *CD24* mRNA expression (Figure 1A) that was over 1000-fold lower in RasV12 cells compared to control cells (Figure 1B).

Similarly, analysis of CD24 surface protein by flow cytometry showed a statistically significant reduction in the percentage of CD24^+^ cells in RasV12 population compared to control cells (Figures 1C,D). Therefore, we can conclude that the reduction of expression is due to the majority of RasV12 cells losing CD24 expression entirely. Together these data show that constitutively active Ras significantly downregulates CD24 at both the mRNA and surface protein expression levels. Residual levels of CD24 are due to a small portion of the population retaining surface expression.

### Ras-mediated repression of *CD24* at the level of the promoter

**Figure 2:**
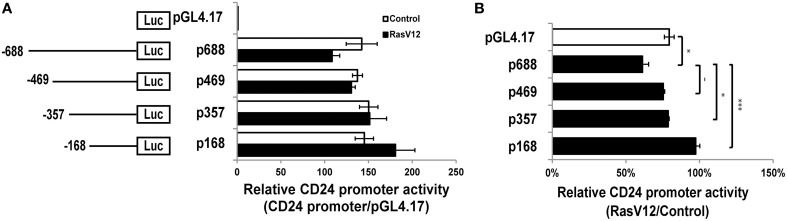
**RasV12 represses ***CD24*** promoter activity. (A)** Relative promoter activity in vector control and RasV12 is shown compared to promoterless vector (pGL4.17). Schematic diagram of promoter deletion constructs are shown on the left. Promoter length is indicated by the position upstream of the TSS. **(B)** Relative promoter activity in RasV12/Control cells for each reporter construct. Significant differences were determined by One-Way ANOVA with Tukey Honest Significant Difference *post-hoc* analysis, *n* = 3, ^−^*P* < 0.1; ^*^P < 0.05; ^***^P < 0.001, all data are shown as mean ± s.e.m.

We next analyzed the activity of the *CD24* promoter region comprising the 688 nucleotides upstream of the TSS (p688) (Pass et al., 1998). We found that this region is active in both RasV12 and control cells compared to the promoterless control (Figure 2A). However, the activity was reduced in RasV12 cells compared to control cells. To determine if this difference was statistically significant, we analyzed the relative activity in RasV12 vs. control cells in comparison to the promoterless vector, which represents basal activity levels in the two cell types (Figure 2B). We found that there was a significant repression of the promoter activity in the RasV12 cells. To further narrow down the responsive region, we analyzed a series of *CD24* promoter deletion mutants (Figures 2A,B). We found the relative promoter activity in RasV12 compared to control cells increased in each deletion mutant compared to the full promoter. This increase approached statistical significance with the p469 promoter region (−469 to −1) and reached significance with the p357 promoter region (−357 to −1). The relative activity was further increased when the promoter included only the region from −168 to −1 from the TSS (p168). Therefore, both the 112 bp region from −469 to −357 and the 189 bp region from −357 to −168 contain repressive elements that are regulated by RasV12. However, the sequence within −357 to −168 promoter region appears to contribute more to the suppression of *CD24* than the sequence within the −469 to −357 region.

### Activation of either the Raf or the PI3K pathway is sufficient to downregulate CD24 expression

Since we observed that CD24 mRNA and protein expression are downregulated by Ras, we next asked which pathways downstream of Ras are sufficient to suppress CD24 expression. Ras can activate three major pathways, the Raf, RalGDS, and PI3K pathways, all of which contribute to the fully transformed phenotype of cancer cells (Hamad et al., 2002). We made use of NIH/3T3 cells expressing the Ras effector mutants RasV12G37, RasV12S35, and RasV12C40 (White et al., 1995; Khosravi-Far et al., 1996; Webb et al., 1998), in which only one pathway is activated. Specifically, RasV12G37 activates only RalGDS but not PI3K or Raf. In a similar manner, RasV12S35 selectively activates the Raf pathway, and RasV12C40 selectively activates the PI3K pathway.

**Figure 3:**
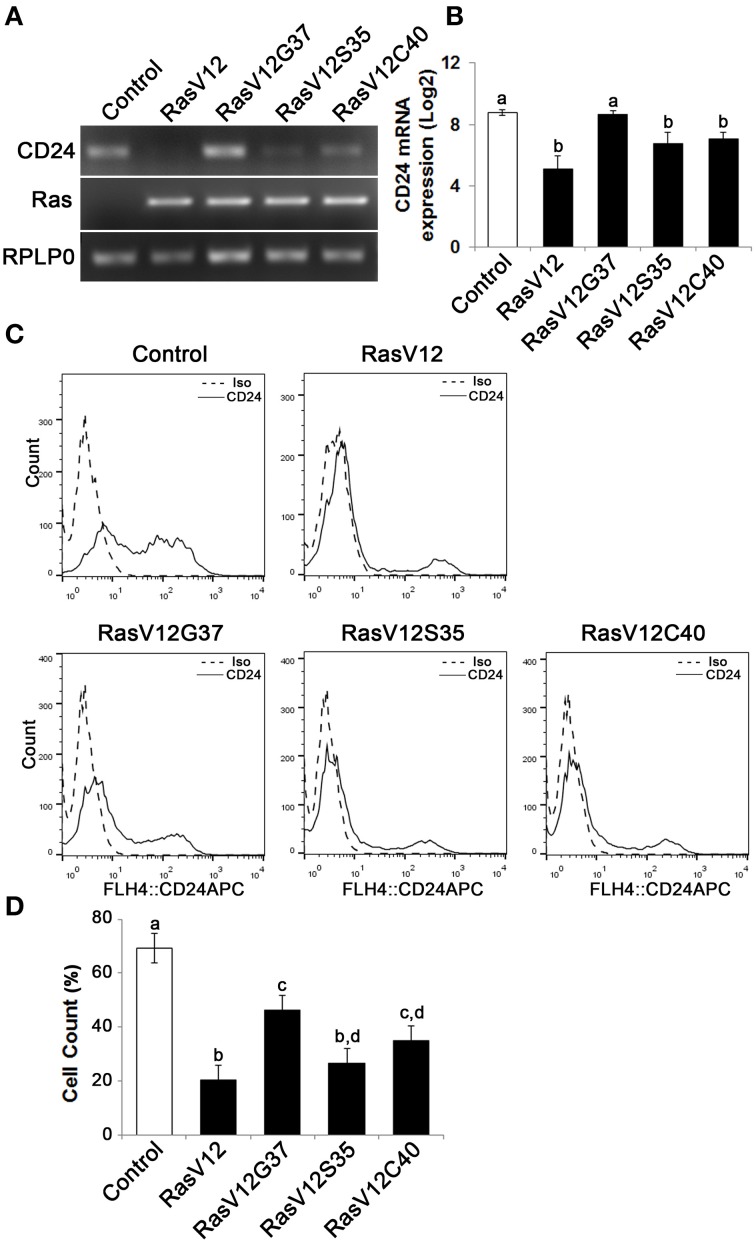
**The Raf and PI3K pathways downregulate ***CD24*** mRNA expression while the Raf pathway is majorly responsible for downregulation of CD24 surface protein expression. (A)** *CD24* mRNA expression in Control, RasV12, RasV12G37, RasV12S35, and RasV12C40 cells was determined by RT-PCR. *H*-*Ras* mRNA expression was used to verify expression of ectopic Ras. *RPLP0* was used as the loading control. **(B)** RT-qPCR was used to quantify *CD24* mRNA expression with *RPLP0* used as the normalization control. **(C)** Surface CD24 protein was determined by flow cytometry. One representative histogram of isotype (Iso) and CD24-stained cells is shown. **(D)** Quantification of CD24 surface protein expression as mean ± s.e.m percentage of CD24^+^ cells. Significance was determined by One-Way ANOVA with Tukey Honest Significant Difference analysis, *n* = 4, different lower case letters indicating different groups at *P* < 0.01.

We observed a downregulation in *CD24* mRNA expression in RasV12S35 and RasV12C40 effector mutants compared to control cells by RT-PCR (Figure 3A). In contrast, the RasV12G37 cells had similar level of *CD24* mRNA expression as the control cells (Figure 3A). Quantitative analysis of *CD24* mRNA by RT-qPCR revealed the change in *CD24* mRNA expression levels in RasV12S35 and RasV12C40 cells to be statistically significant when compared to control cells and RasV12G37 but not different from RasV12 cells (Figure 3B).

We then determined if CD24 surface protein expression was also affected in Ras effector mutant cells (Figure 3C). We found that there was a significant and substantial reduction in the percentage of cells expressing CD24 surface protein in RasV12 and RasV12S35 cells (Figure 3D). RasV12G37 and RasV12C40 expression had an intermediate effect on reducing the percentage of cells expressing CD24, compared to control cells. Therefore, activation of the Raf kinase pathway, the PI3K pathway, or the RalGDS pathway downstream of Ras are sufficient to decrease the CD24^+^ population. However, activation of the Raf pathway was sufficient to decrease the CD24^+^ population to the same low level as RasV12 suggesting that the Raf pathway is the major regulator of CD24 expression in these cells. Moreover, either Raf or PI3K could decrease both CD24 mRNA and surface expression while RalGDS only partially affected surface expression.

### Inhibition of MEK or PI3K does not fully restore *CD24* mRNA expression

**Figure 4:**
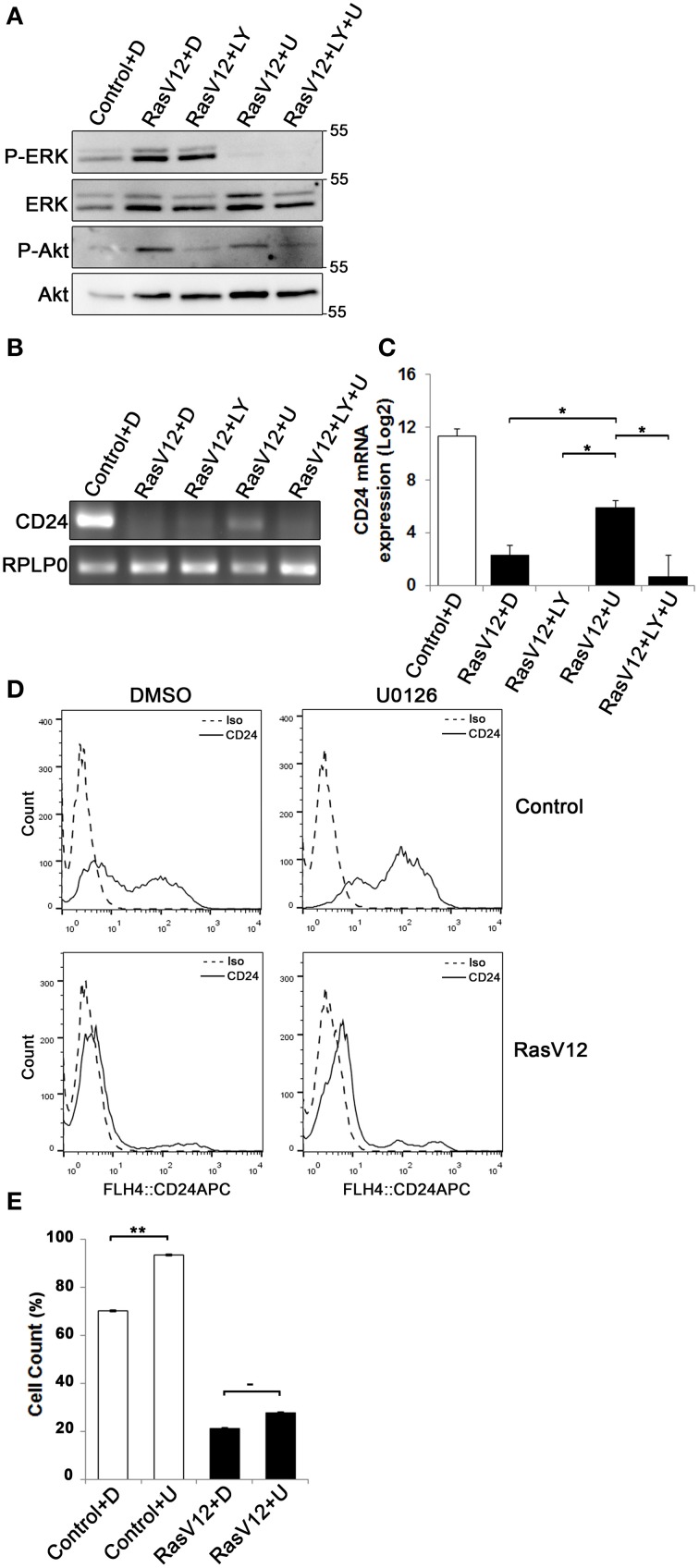
**Inhibition of the Raf/MEK/ERK pathway is sufficient to partially restore ***CD24*** mRNA but not protein expression in RasV12 cells**. **(A–C)** RasV12 cells were treated for 16 h with DMSO **(D)**, or U0126 (U) and/or LY294002 (LY). **(A)** Western blot analysis was performed to detect phosphorylated ERK (P-ERK), and phosphorylated Akt (P-Akt). Total ERK and total Akt were used as loading controls. Molecular mass standards are shown in the right of each image. One representative experiment from three replicates is shown. *CD24* mRNA expression in Control and RasV12 cells was determined by **(B)** RT-PCR and **(C)** RT-qPCR. *RPLP0* was used as the loading and normalization control. Significance was determined by One-Way ANOVA with Tukey Honest Significant Difference *post-hoc* analysis, ^*^P < 0.05. **(D)** Surface CD24 protein was determined by flow cytometry with Control or RasV12 cells treated for 24 h as above. One representative histogram of isotype (Iso) and CD24-stained cells is shown. **(E)** Quantification of CD24 surface protein expression as mean ± s.e.m percentage of CD24^+^ cells. Significance was determined by student's *t*-test, *n* = 4, ^−^*P* < 0.1; ^**^P < 0.01.

Since we observed that both CD24 mRNA and protein is significantly and substantially downregulated by the Raf and PI3K pathways we next determined if inhibition of either or both of these pathways could restore *CD24* expression in RasV12 cells at the mRNA level. The major downstream effectors of Raf are the MEK1/2 kinases (Gollob et al., 2006), which can be inhibited specifically with the chemical inhibitor U0126 (Davies et al., 2000). PI3K can be directly inhibited using LY294002 (Davies et al., 2000; Xue et al., 2007). We evaluated the inhibition of Raf/MEK/ERK and PI3K/Akt pathways using western blot analysis of phosphorylated ERK (P-ERK) and phosphorylated Akt (P-Akt), respectively (Figure 4A). We found that RasV12 cells treated with U0126 had reduced phosphorylation of ERK with no effect on Akt phosphorylation. Similarly, RasV12 cells treated with LY294002 had reduced phosphorylation of Akt with no effect on ERK phosphorylation. Treatment with both U0126 and LY294002 inhibited phosphorylation of both ERK and Akt. We found that treatment of RasV12 cells with U0126 had a significant 13-fold increase in *CD24* mRNA expression (Figures 4B,C). Surprisingly, treatment with LY294002 alone or in combination with U0126 suppressed *CD24* mRNA expression to below the levels seen in RasV12 cells (Figures 4B,C).

Since U0126 increased *CD24* mRNA levels, we next determined the effect of U0126 on CD24 surface protein in control cells and RasV12 cells. Unexpectedly, we found that U0126 increased the percentage of CD24^+^ cells in the control cell population (Figures 4D,E). In contrast, there was a modest but not statistically significant increase in the percentage of CD24^+^ RasV12 cells treated with U0126 (Figures 4D,E). Therefore, even though activation of the Raf pathway is sufficient to decrease CD24 mRNA and protein, inhibition of oncogenic Raf/MEK signaling does not restore CD24 expression to the level of control cells at the mRNA or protein level.

### Inhibition of Raf partially restores *CD24* cell surface protein expression in cells expressing oncogenic Ras but not control cells

**Figure 5:**
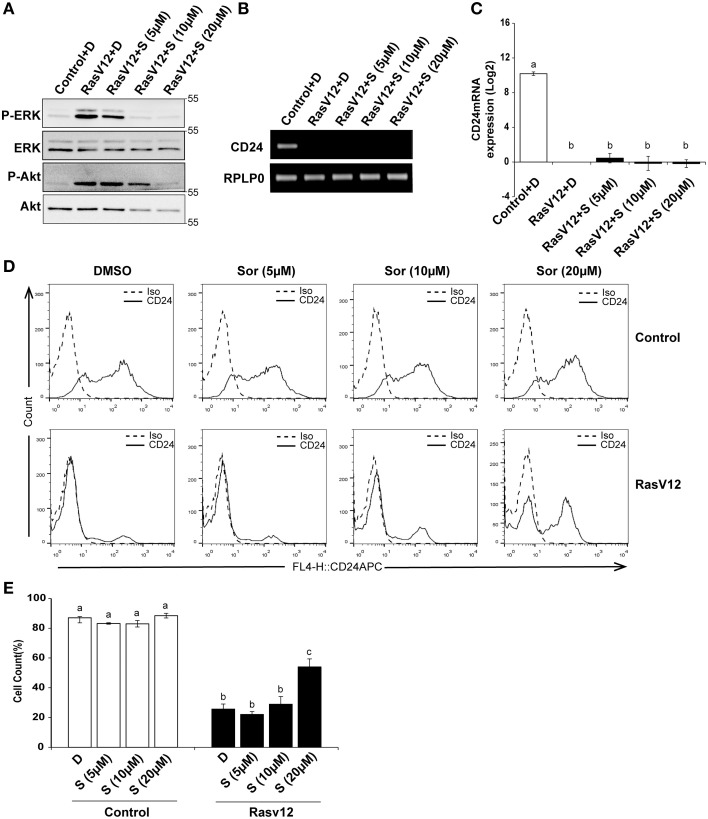
**Inhibition of Raf is sufficient to increase CD24 cell surface protein but not mRNA expression in RasV12 cells**. **(A–C)** Rasv12 cells were treated for 16 h with DMSO **(D)** or 5, 10, and 20 μM sorafenib (S). **(A)** Western blot analysis was performed as in Figure 4. One representative experiment from three replicates is shown. *CD24* mRNA expression in Control and RasV12 was determined by **(B)** RT-PCR and **(C)** RT-qPCR as in Figure 4. Significance was determined by One-Way ANOVA with Tukey Honest Significant Difference *post-hoc* analysis, *n* = 3. **(D)** Surface CD24 protein was determined by flow cytometry with Control and RasV12 treated for 24 h as above. One representative histogram of isotype (Iso) and CD24-stained cells is shown. **(E)** Quantification of CD24 surface protein expression as mean ± s.e.m percentage of CD24^+^ cells. Significance was determined by One-Way ANOVA with Tukey Honest Significant Difference analysis, *n* = 3, different lower case letters indicate different groups at *P* < 0.001.

Since we observed that inhibition of MEK partially restored *CD24* mRNA with no significant effect on protein levels we next determined if inhibition of Raf directly could restore CD24 expression levels. Raf is the major downstream target of Ras and can be directly inhibited by sorafenib, which does not inhibit MEK or ERK (Wilhelm et al., 2004). We evaluated the inhibition of Raf/MEK/ERK pathway by sorafenib at different concentrations using western blot analysis of phosphorylated ERK (P-ERK) and phosphorylated Akt (P-Akt) as measures of efficacy and specificity (Figure 5A). We found that sorafenib reduced ERK phosphorylation in RasV12 cells at all concentrations examined. We also found that both phosphorylated and total Akt was reduced with 10 and 20 μM sorafenib. Sorafenib is known to inhibit additional kinases such as EGFR, PDGFR, c-Kit and FLT-3 (Wilhelm et al., 2004; Adnane et al., 2006), therefore it is not surprising to observe inhibition of additional pathways with this inhibitor. However, we found that treatment of RasV12 cells with sorafenib had no effect on *CD24* mRNA expression (Figures 5B,C).

We next determined the effect of Raf inhibition by sorafenib on CD24 surface protein in control cells and RasV12 cells. We found that treatment with 20 μM but not 5 or 10 μM sorafenib significantly increased the percentage of CD24^+^ cells within the RasV12 cell population (Figures 5D,E). In contrast to MEK inhibition, there was no change in the percentage of CD24^+^ cells in the control cells after treatment with sorafenib (Figures 5D,E). Therefore, inhibition of Raf significantly increases the proportion of CD24^+^ cells in the absence of changes at mRNA level, but only in cells expressing oncogenic Ras.

### Inhibition of PI3K does not synergize with Raf inhibition to affect *CD24* mRNA or surface protein expression

**Figure 6:**
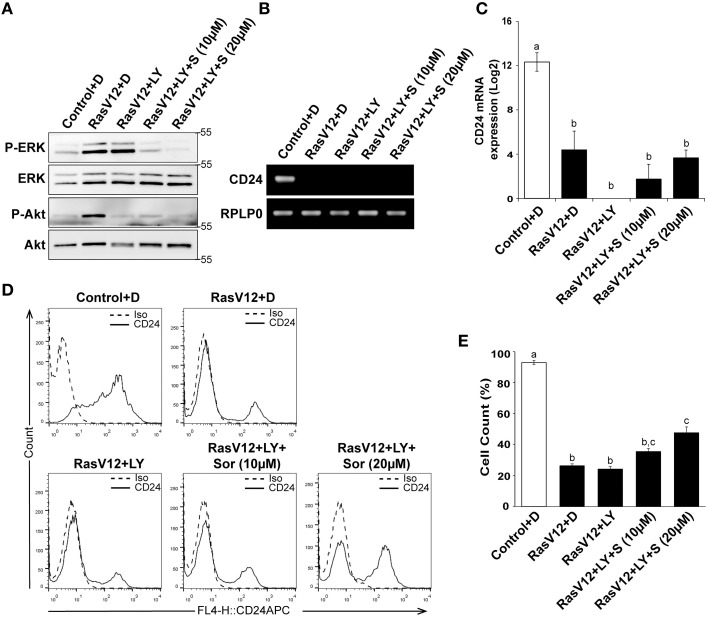
**Inhibition of PI3K did not alter the Raf-mediated inhibition of CD24 expression**. **(A–C)** Rasv12 cells were treated for 16 h with DMSO **(D)**, 100 μM LY294002 (LY) and/or 10 or 20 μM sorafenib (S/Sor) with LY. **(A)** Western blot analysis was performed as in Figure 4. One representative experiment from three replicates is shown. *CD24* mRNA expression in Control and RasV12 was determined by **(B)** RT-PCR and **(C)** RT-qPCR as in Figure 4. Significance was determined by One-Way ANOVA with Tukey Honest Significant Difference *post-hoc* analysis, *n* = 3, ^a, b^*P* < 0.01. **(D)** Surface CD24 protein was determined by flow cytometry with Control and RasV12 for 24 h as above. One representative histogram of isotype (Iso) and CD24-stained cells is shown. **(E)** Quantification of CD24 surface protein expression as mean ± s.e.m percentage of CD24^+^ cells. Significance was determined by One-Way ANOVA with Tukey Honest Significant Difference analysis, *n* = 3, different lower case letters indicating different groups at *P* < 0.001.

We next determined if inhibition of both PI3K and Raf together could further restore CD24 expression in RasV12 cells at the mRNA and surface protein levels. We evaluated the inhibition of PI3K/Akt and Raf/MEK/ERK pathways using western blot analysis of phosphorylated ERK (P-ERK) and phosphorylated Akt (P-Akt), respectively, as previously discussed (Figure 6A). Similar to our previous observations, we found that treatment of RasV12 with LY294002 reduced phosphorylation of Akt with no effect on phosphorylation of ERK. Treatment with both LY294002 and sorafenib inhibited phosphorylation of both ERK and Akt, as expected. We found that treatment with both LY294002 and sorafenib did not increase *CD24* mRNA expression (Figures 6B,C).

We found that treatment of RasV12 cells with LY294002 alone did not affect the percentage of CD24^+^ cells in either control or RasV12 cells (Figures 6D,E). Moreover, addition of LY294002 did not affect the sorafenib-induced increase in the percentage of CD24+ cells (Figure 6E). Together, these data indicate that even though activation of either the PI3K or Raf pathway is sufficient to decrease CD24 mRNA and protein expression, inhibition of both PI3K and Raf together is not sufficient to restore CD24 surface protein or mRNA expression.

## Discussion

**Figure 7:**
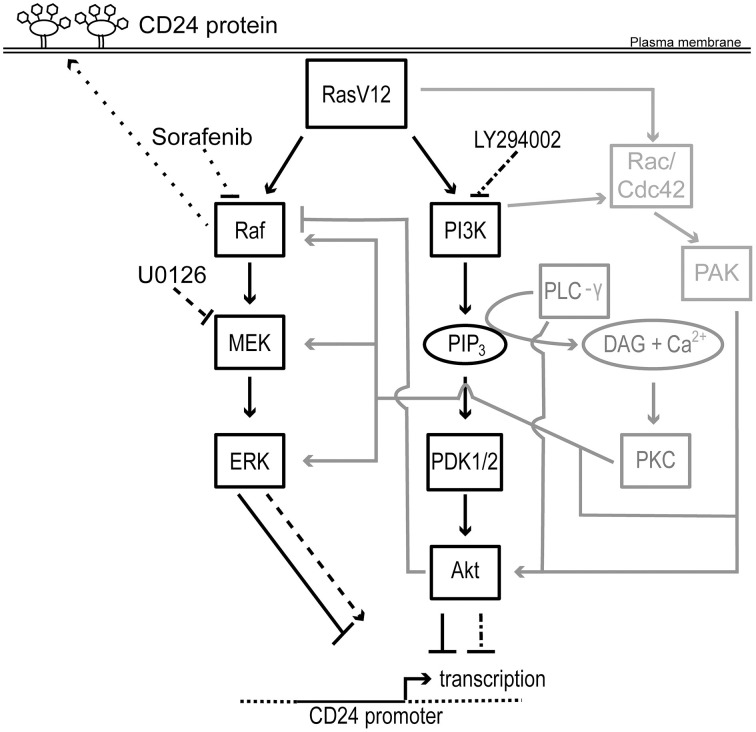
**Schematic representation of the regulation of CD24 by Ras/Raf and Ras/PI3K pathways**. Rectangles represent proteins and ovals represent lipids and second messengers. Protein activation is indicated by solid arrows and inhibition indicated by solid lines with blunt ends. Potential mechanisms for cross-talk are shown in gray. The effect of U0126 and LY294002 on MEK and PI3K and on *CD24* transcription is indicated by dashed lines and dash dot dash lines, respectively. The effect of sorafenib on Raf and on CD24 surface protein expression is indicated by dotted lines. The *CD24* promoter is indicated with a solid line flanked by genomic DNA depicted with a dotted line. The transcription start site is indicated by a bent arrow. Abbreviations not in the main text: PLC-γ, phospholipase C- γ; PIP_3_, phosphotidylinositol-3,4,5-trisphosphate; DAG, diacylglycerol; Cdc42, cell division cycle 42.

Here, we have demonstrated that expression of oncogenic Ras is sufficient to directly downregulate expression of CD24 at the mRNA and protein levels as well as repress promoter activity. Moreover, activation of either the Raf or PI3K pathway is sufficient to downregulate CD24 expression at both the mRNA and protein levels (Figure 7). Surprisingly, inhibition of the Raf pathway, at the level of MEK or Raf, or the PI3K pathway, at the level of PI3K, either separately or together, was not sufficient to fully restore CD24 expression in RasV12 cells. However, inhibition of Raf directly was able to partially restore CD24 surface protein expression without affecting mRNA levels.

Our experimental analysis of the *CD24* promoter region revealed that repression of *CD24* promoter activity by oncogenic Ras was primarily mediated by the 189 bp region between −168 and −357. In addition, a 112 bp region between −357 and −469 also appears to contribute to this repression. We did not observe any regulation of the previously identified negative regulatory region (−983 to −1996) (Pass et al., 1998) by oncogenic Ras (data not shown). Therefore, we have identified a novel negative regulatory region between −168 and −357 that may act independently or in cooperation with additional elements between −357 and −469 to regulate *CD24* transcription in response to oncogenic Ras.

Computational analysis of the 112 bp region revealed more than 300 potential transcription factor binding sites including five sites that can bind TWIST transcription factors. However, deletion of these sites did not affect the repression of *CD24* promoter by Ras (data not shown). While regulation of the *CD24* promoter by methylation has been reported in diseased conjunctiva (Riau et al., 2011) and glioblastoma cell lines (Fukushima et al., 2007), there was no evidence of promoter methylation of *CD24* in breast cancer cell lines or in patient tumors (Kagara et al., 2012). Therefore, further work is necessary to identify the precise regulatory mechanism used by Ras to repress *CD24* transcription.

To narrow down which of the Ras-activated pathways represses CD24 expression we made use of the Ras effector mutants, which activate select downstream pathways (White et al., 1995; Khosravi-Far et al., 1996; Webb et al., 1998). We found that RasV12S35 cells, in which the Raf pathway is constitutively active, and RasV12C40 cells, in which the PI3K pathway is constitutively active, had reduced *CD24* mRNA expression compared to control cells. In contrast, the RasV12G37 cells retained statistically similar mRNA levels to control cells. With respect to surface protein levels, we found that CD24 expression was downregulated by all the Ras effector mutants, including RasV12G37, when compared to control cells. However, the surface expression of CD24 was statistically higher in RasV12G37 and RasV12C40 compared to RasV12 cells. Strikingly, the surface expression of CD24 in RasV12S35 cells was downregulated to a statistically similar level as RasV12. Therefore, together these data suggest that activation of either the PI3K or the Raf pathway is sufficient to decrease *CD24* mRNA expression but the Raf pathway is the major repressor of CD24 expression at both the mRNA and protein levels.

Unexpectedly, when we performed the complementary loss-of-function experiments using either the MEK inhibitor U0126 or the Raf inhibitor sorafenib in the presence or absence of the PI3K inhibitor LY294002, we were unable to restore *CD24* mRNA expression to the level of the control cells. Treatment with U0126 caused a 13-fold increase, which is still 100-fold lower than the mRNA levels present in control cells, and therefore may be responsible for the modest but not statistically significant increase in CD24 surface expression. In contrast, sorafenib treatment did not alter *CD24* mRNA levels. Inhibition of PI3K alone did not alter *CD24* mRNA levels but in combination with MEK inhibition prevented the U0126-mediated increase in *CD24* mRNA expression. Thus, these data suggest that the inhibition of the PI3K pathway could potentially activate a repressor or inhibit an activator of *CD24* mRNA expression to block the effects of U0126 treatment. Consistent with this hypothesis, significant cross-talk between the PI3K and Raf pathways has been shown, including the ability of Akt to inhibit Raf. As we did not observe an increase in ERK phosphorylation, the possible relief of Akt-mediated inhibition of Raf did not override the U0126-mediated block in MEK activation. Thus, inhibition of the PI3K pathway may relieve the Akt-mediated inhibition of Raf and promote Raf-mediated suppression of CD24 (Figure 7).

Interestingly, sorafenib treatment significantly increased the proportion of CD24^+^ RasV12 cells while U0126 treatment significantly increased CD24^+^ cells only in the control cell population. This suggests that regulation at the level of Raf regulates CD24 expression in oncogenic conditions while MEK can regulate CD24 expression in response to basal growth conditions. In both situations ERK phosphorylation is equally inhibited, therefore the regulation of CD24 surface expression cannot be downstream of ERK activation. Together, these data demonstrate that the regulation of CD24 surface protein expression is regulated in a Raf-dependent, MEK-independent manner. Furthermore, these data suggest that additional pathways remain activated in the presence of the Raf, MEK, or PI3K inhibition that can continue to repress CD24 expression. For example, as depicted in Figure 7, both p21 protein (Cdc42/Rac)-activated kinase (PAK) and Protein Kinase C (PKC) can positively regulate the Raf/MEK/ERK pathway at multiple levels to bypass Raf or MEK inhibition (Kaga et al., 1998; Jimenez et al., 2000; Aksamitiene et al., 2012).

These inhibitor experiments as well as our observation that there is a reduction in CD24 cell surface protein expression but not mRNA expression in RasV12G37 cells strongly suggest that the downregulation of CD24 mRNA and surface expression are not mediated by the same mechanisms. CD24 has substantial post-translational modifications, including protein cleavage, addition of the GPI-anchor, and major O- and N-linked glycosylations that occur prior to surface expression (Fang et al., 2010). Thus, the Ras/Raf or the Ras/Ral pathways may also regulate post-translational modification or trafficking. The interaction of Raf-1 with the actin cytoskeleton has been shown to be necessary for efficient activation of MEK/ERK (Wang et al., 2013), but no Raf-mediated MEK-independent regulation of protein trafficking has been reported. On the other hand, Ral is well-known to regulate vesicle trafficking (Feig, 2003). While Ral can be activated in a Ras-independent manner (Linnemann et al., 2002), it is not known if Ral can be activated in a Raf-dependent manner. Alternatively, alteration of GPI-anchor biosynthesis by Ras as, previously identified in *Saccharomyces cerevisiae* (Sobering et al., 2004), may be responsible for altering CD24 surface expression. Therefore, future work will be necessary to unravel the precise mechanism that regulates CD24 surface protein expression by the Ras/Raf pathway.

As described earlier, high CD24 expression is associated with promoting proliferation in lung, prostate, and colorectal cancer (Kristiansen et al., 2004), while low CD24 expression is associated with proliferation in BCSCs (Sheridan et al., 2006). This paradoxical relationship does not yet have a mechanistic explanation but may be related to the cell type of origin. Our model system clearly recapitulates the BCSC phenotype of oncogenic Ras and transformation being associated with low levels of CD24 (Marcato et al., 2009).

CD24 expression is dynamically regulated throughout the development of B and T lymphocytes, dendritic cells, neurons, and adipocytes (Nathan et al., 1986; Smith et al., 2015; Tan et al., 2015). Moreover, CD24 expression in some normal cell types is associated with proliferation or differentiation (Li et al., 2004; Smith et al., 2015), while in others it is associated with apoptosis (Suzuki et al., 2001). Therefore, transformation of a particular cell type at a particular cell stage may dictate if CD24 promotes or inhibits proliferation. Previously, it was found that overexpression of oncogenic H-Ras as the last step in the sequential transformation of primary human mammary epithelial cells or in immortalized MCF10A cells resulted in an epithelial to mesenchymal transition (EMT) concomitant with the downregulation of CD24 surface expression (Morel et al., 2008). Here we have found that expression of oncogenic Ras in mouse embryonic fibroblasts, which are already mesenchymal, represses CD24 mRNA and protein expression, and promoter activity. Therefore, it may be the mesenchymal phenotype that predisposes a cell to lose CD24 expression in response to Ras transformation while other cell types will gain or retain CD24 expression dependent on their epithelial vs. mesenchymal status.

Overall, understanding the regulation of CD24 expression by oncogenic Ras may promote development of therapeutic strategies to induce BCSCs to gain CD24 expression. This strategy would decrease the percentage of BCSCs in the tumor and therefore reduce the ability of the tumor to be radiation-resistant or initiate secondary tumor formation. We have demonstrated that CD24 expression is directly suppressed by oncogenic Ras, however, while inhibition of the MEK was sufficient to partially restore *CD24* expression at the mRNA level, inhibition of Raf was necessary to increase CD24^+^ cells. Thus, these data clearly demonstrate that the oncogenic Ras-mediated suppression of CD24 expression is regulated at multiple levels. However, as sorafenib clearly increases the population of CD24^+^ cells, this clinically relevant therapeutic should be considered in situations where shifting the population of CD24^−^ toward CD24^+^ would be beneficial.

## Author contributions

NP designed, performed, and analyzed the experiments, and wrote the manuscript. DA analyzed the promoter region and edited the manuscript. SC conceived the study, designed the experiments and wrote the manuscript. All authors read and approved the final manuscript.

### Conflict of interest statement

The authors declare that the research was conducted in the absence of any commercial or financial relationships that could be construed as a potential conflict of interest.

## Abbreviations

BCSCs: Breast cancer stem cells
ER: estrogen receptor
PI3K: phosphoinositide-3-kinase
ERK: Extracellular signal-regulated kinases
PLD: phospholipase D
PDK1: phosphoinositide-dependent kinase 1
PDK2: phosphoinositide-dependent kinase 2
TSS: transcriptional start site.
PKC: protein kinase C
PAK: p21-activated kinase.
